# Regional Variations in Alirocumab Dosing Patterns in Patients with Heterozygous Familial Hypercholesterolemia During an Open-Label Extension Study

**DOI:** 10.1007/s10557-020-06984-0

**Published:** 2020-05-04

**Authors:** Gisle Langslet, G. Kees Hovingh, John R. Guyton, Marie T. Baccara-Dinet, Alexia Letierce, Garen Manvelian, Michel Farnier

**Affiliations:** 1 Lipid Clinic, Oslo University Hospital, Aker Sykehus, Bygg 6, Trondheimsveien 235, Postboks 4959 Nydalen, 0424 Oslo, Norway; 2 Academic Medical Center, Amsterdam, The Netherlands; 3 Novo Nordisk AS, Copenhagen, Denmark; 4 Duke University Medical Center, Durham, NC USA; 5 Clinical Development, R&D, Sanofi, Montpellier, France; 6 Biostatistics and Programming, Sanofi, Chilly-Mazarin, France; 7 Regeneron Pharmaceuticals, Inc., Tarrytown, NY USA; 8 Lipid Clinic, Point Médical and Department of Cardiology, CHU Dijon-Bourgogne, Dijon, France

**Keywords:** Alirocumab, Familial hypercholesterolemia, LDL-C, Open-label extension, PCSK9, Dose adjustment

## Abstract

**Purpose:**

During the alirocumab open-label extension study ODYSSEY OLE (open-label extension; NCT01954394), physicians could adjust alirocumab dosing for enrolled patients, who were diagnosed with heterozygous familial hypercholesterolemia (HeFH) and who had completed previous phase III clinical trials with alirocumab. This post hoc analysis evaluated the differences in physician–patient dosing decisions between the regions of Western Europe, Eastern Europe, North America, and the rest of the world (ROW).

**Methods:**

Patients (*n* = 909) who received starting dose alirocumab 75 mg every 2 weeks (Q2W) during ODYSSEY OLE (patients from FH I, FH II, and LONG TERM parent studies) were included. Low-density lipoprotein cholesterol (LDL-C) levels were blinded until week 8; subsequently, LDL-C values were communicated to physicians. From week 12, dose adjustment from 75 to 150 mg Q2W, or vice versa, was possible.

**Results:**

Mean LDL-C values used for the decision to increase dose from 75 to 150 mg Q2W were higher in Eastern Europe (3.7 mmol/L; 144.0 mg/dL) and ROW (3.8 mmol/L; 145.2 mg/dL) compared with Western Europe (3.1 mmol/L; 118.6 mg/dL) and North America (3.3 mmol/L; 126.6 mg/dL). Irrespective of region, the mean LDL-C at the time of decision to maintain at 75 mg Q2W was approximately 1.8 mmol/L (70 mg/dL). During ODYSSEY OLE (median treatment duration of 131.7 weeks), alirocumab was shown to have no unexpected long-term safety concerns.

**Conclusions:**

In this OLE study, the observed variations in clinical treatment decisions suggest that physicians may perceive the severity of HeFH and/or the treatment of HeFH differently depending on their region.

**Electronic supplementary material:**

The online version of this article (10.1007/s10557-020-06984-0) contains supplementary material, which is available to authorized users.

## Introduction

Heterozygous familial hypercholesterolemia (HeFH) is a common genetic disorder characterized by elevated levels of low-density lipoprotein cholesterol (LDL-C) [1–3]. In clinical practice, many patients with HeFH do not reach their LDL-C treatment goals, remaining at high risk of atherosclerotic cardiovascular disease (ASCVD) [4–7]. Patients with HeFH are typically initiated on maximally tolerated statin therapy, with or without ezetimibe. Recent European and American guidelines recommend considering the addition of a proprotein convertase subtilisin/kexin type 9 (PCSK9) inhibitor for patients with HeFH and LDL-C ≥ 100 mg/dL (≥ 2.6 mmol/L) while taking maximally tolerated statin and ezetimibe therapy [5, 7].

Alirocumab, a monoclonal antibody that inhibits PCSK9, was shown in randomized controlled trials to significantly lower levels of LDL-C and other lipids compared with placebo or ezetimibe in patients with and without HeFH [8–11]. Of note, all patients were receiving background statin with or without other lipid-lowering therapies (LLTs). The alirocumab open-label extension study ODYSSEY OLE (open-label extension; NCT01954394) included patients diagnosed with HeFH who had completed previous phase III parent studies, and was designed to assess the long-term safety and efficacy of alirocumab (2.5 years median alirocumab exposure during ODYSSEY OLE) [12]. Alirocumab was administered subcutaneously in one of the two available dosages (75 or 150 mg every 2 weeks [Q2W]), and, unique within the ODYSSEY alirocumab clinical development program, with dose adjustment per physician’s clinical judgment applied to physician–patient shared decision-making.

The aim of this post hoc analysis was to examine the regional differences in dosing decisions during ODYSSEY OLE, which are guided by both local and international clinical guidelines.

## Methods

### Study Design

The ODYSSEY OLE study design has been reported previously [12]. In short, patients with HeFH receiving maximally tolerated dose statins (± other LLTs) were eligible to enter ODYSSEY OLE if they had completed one of four phase III double-blind parent studies. The current analysis only includes data from patients who received alirocumab 75 mg Q2W at entry to ODYSSEY OLE (patients from FH I [9], FH II [9], and LONG TERM [10] studies). For patients from FH I and FH II, the end of the double-blind treatment period corresponded with the start of the ODYSSEY OLE study. Patients who participated in LONG TERM had an 8-week off-treatment wash-out period prior to the start of ODYSSEY OLE as per protocol. All patients received alirocumab 75 mg Q2W at ODYSSEY OLE entry irrespective of treatment (alirocumab or placebo) or alirocumab dose received during the parent studies (first patient enrolled in December 2013, with study completion in June 2017).

The ODYSSEY OLE study comprised a treatment period of up to 168 weeks and an additional 8-week follow-up period for patients who completed the study or discontinued early. LDL-C levels were blinded to the patient and physician from day 1 until week 8; after week 8, LDL-C values were communicated to physicians. From week 12, physicians could adjust the dose from alirocumab 75 to 150 mg Q2W, or vice versa, based on their clinical judgment, the patient’s LDL-C level, and patient preference. During ODYSSEY OLE, patients received, as much as possible, the same stable maximally tolerated statin dose ± other LLTs as during the parent study.

The ODYSSEY OLE study protocol was approved by the appropriate independent review board/ethics committee, and all investigators conducted the study in accordance with ethical guidelines based on the Declaration of Helsinki as well as with the International Conference on Harmonization Guidelines for Good Clinical Practice and applicable regulatory requirements. All patients provided written informed consent.

### Outcome Measures

**Fig. 1:**
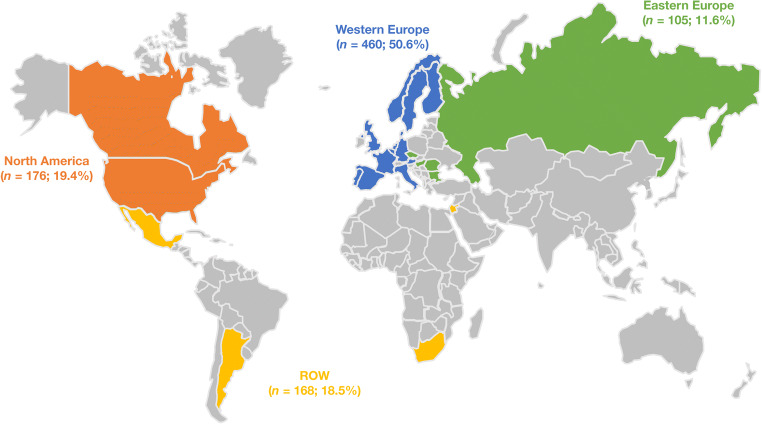
Regional distribution of patients in ODYSSEY OLE included in current analysis (safety population). North America: Canada and the USA. Western Europe: Austria, Belgium, Denmark, Finland, France, Germany, Italy, the Netherlands, Norway, Portugal, Spain, Sweden, and the UK. Eastern Europe: Bulgaria, Czech Republic, Hungary, Romania, and the Russian Federation. ROW: Argentina, Israel, Mexico, and South Africa. ROW, rest of world.

The regional differences in the utilization of the alirocumab dosing strategy was assessed by determining the regional variations in the number and proportion (percent) of patients who were maintained on 75 mg Q2W, those who received dose increase from 75 to 150 mg Q2W, and those who received subsequent dose decrease from 150 to 75 mg Q2W during ODYSSEY OLE. Alirocumab dose-adjustment decisions were also analyzed according to ASCVD status at baseline; ASCVD was defined as coronary heart disease (CHD), ischemic stroke, or peripheral artery disease. Patient adherence to alirocumab treatment was assessed according to patients’ diary data; percent adherence was defined as 100-(percent of days with below-planned dosing + percent of days with above-planned dosing). The long-term efficacy of alirocumab was assessed by region, including both absolute and percentage changes from baseline of the parent studies in calculated LDL-C and other lipid parameters. Efficacy was also assessed by evaluating the proportion of patients achieving LDL-C < 100 mg/dL or < 70 mg/dL, and the proportion of patients with either LDL-C < 70 mg/dL and/or ≥ 50% reduction from parent–study baseline (if LDL-C ≥ 70 mg/dL). Safety and tolerability parameters were also assessed throughout the study. Treatment-emergent adverse events were defined as those occurring from the first to the lazst dose of alirocumab in ODYSSEY OLE plus 70 days. Data were assessed in four regions: Western Europe, North America, Eastern Europe, and the rest of the world (ROW; Fig. 1). Each region is composed of multiple countries; for most countries, there were multiple investigators involved in the ODYSSEY OLE trial (refer to Supplementary Material provided by Farnier and colleagues [12]).

### Statistical Analyses

Most current analyses were performed on the safety population, defined as all patients who received at least one dose or partial dose of alirocumab during ODYSSEY OLE. Dose-adjustment decisions are derived from the system managing the study drug dispensation: the investigator had to contact the system to manage the dose, which generally occurred every 12 weeks or at any time if required. The proportions of patients with any dose decision (maintenance at 75 mg Q2W, increase to 150 mg Q2W, maintenance at 150 mg Q2W, or decrease from 150 to 75 mg Q2W) during the study are calculated using the appropriate denominator (e.g., maintenance at 150 mg Q2W is assessed in patients receiving 150 mg Q2W). Efficacy analyses were performed on the modified intention-to-treat population, which included patients who received at least one dose or partial dose of the study drug during OLE, who had baseline LDL-C data from the parent study, and who had at least one other LDL-C value available during the appropriate analysis window from the period of first study drug injection in ODYSSEY OLE and up to 3 weeks after the last study drug injection. As this was a post hoc analysis, safety outcomes, the assessment of dose-adjustment decisions, and efficacy parameters were analyzed by descriptive statistics only.

## Results

### Patient Characteristics

The regional distribution of all patients (*n =* 909; safety population) is presented in Fig. 1; half of all participants (*n =* 460; 50.6%) were from Western Europe. Baseline characteristics for these patients are shown in Table 1. Baseline mean LDL-C levels were higher in ROW and North America (3.5 and 3.4 mmol/L; 133.4 and 132.1 mg/dL, respectively) compared with Western Europe and Eastern Europe (3.1 and 2.4 mmol/L; 120.2 and 93.6 mg/dL, respectively). As reported previously, nearly all patients (99.2%) were receiving statins at baseline [12]. Overall, 46.9% of patients had ASCVD and 50.1% were at very high cardiovascular risk (defined as patients with CHD or CHD risk equivalents, according to European Society of Cardiology [ESC]/European Atherosclerosis Society [EAS] 2016 guidelines) [5].

**Table 1:** Baseline demographics, medical history, and lipid parameters at OLE baseline (safety population)

Parameter	North America (*n =* 176)	Western Europe (*n =* 460)	Eastern Europe (*n =* 105)	ROW (*n =* 168)	All (*n =* 909)
Age, years, mean (SD)	53.1 (12.3)	54.7 (11.3)	54.7 (12.7)	55.9 (11.6)	54.6 (11.7)
Male, *n* (%)	111 (63.1)	267 (58.0)	56 (53.3)	76 (45.2)	510 (56.1)
Race, *n* (%)					
White	160 (90.9)	454 (98.7)	105 (100.0)	148 (88.1)	867 (95.4)
Black or African-American	3 (1.7)	0 (0.0)	0 (0.0)	0 (0.0)	3 (0.3)
Asian	3 (1.7)	4 (0.9)	0 (0.0)	1 (0.6)	8 (0.9)
American Indian or Alaska Native	2 (1.1)	0 (0.0)	0 (0.0)	1 (0.6)	3 (0.3)
Native Hawaiian or Other Pacific Islander	1 (0.6)	0 (0.0)	0 (0.0)	0 (0.0)	1 (0.1)
Other	7 (4.0)	2 (0.4)	0 (0.0)	18 (10.7)	27 (3.0)
Ethnicity, *n* (%)					
Hispanic or Latino	12 (6.8)	17 (3.7)	0 (0.0)	5 (3.0)	34 (3.7)
Not Hispanic or Latino	164 (93.2)	437 (95.0)	105 (100.0)	163 (97.0)	869 (95.6)
Unknown	0 (0.0)	6 (1.3)	0 (0.0)	0 (0.0)	6 (0.7)
BMI, kg/m^2^, mean (SD)	29.8 (5.3)	28.8 (4.7)	29.1 (4.5)	30.3 (6.0)	29.3 (5.1)
ASCVD,^a^*n* (%)	93 (52.8)	209 (45.4)	49 (46.7)	75 (44.6)	426 (46.9)
CHD^b^	90 (51.1)	200 (43.5)	42 (40.0)	73 (43.5)	405 (44.6)
Very high CV risk,^c^*n* (%)	99 (56.3)	221 (48.0)	52 (49.5)	83 (49.4)	455 (50.1)
High CV risk,^d^*n* (%)zz	77 (43.8)	239 (52.0)	53 (50.5)	85 (50.6)	454 (49.9)
Lipid parameters (mmol/L)					
Calculated LDL-C, mean (SD)	3.4 (1.7)	3.1 (1.7)	2.4 (1.6)	3.5 (1.8)	3.2 (1.7)
Non-HDL-C, mean (SD)	4.2 (1.9)	3.7 (1.9)	3.2 (2.0)	4.2 (1.9)	3.9 (1.9)
Total cholesterol, mean (SD)	5.4 (1.8)	5.1 (1.8)	4.7 (2.0)	5.5 (1.8)	5.2 (1.9)
HDL-C, mean (SD)	1.3 (0.4)	1.3 (0.4)	1.5 (0.4)	1.3 (0.4)	1.3 (0.4)
Triglycerides, median (Q1:Q3)	1.3 (1.0:2.1)	1.2 (0.9:1.6)	1.3 (1.0:1.6)	1.5 (1.0:2.1)	1.3 (0.9:1.8)
Lp(a), mg/dL, median (Q1:Q3)	34.0 (12.0:80.0)	24.0 (7.0:71.5)	8.0 (3.0:36.0)	21.0 (9.0:50.0)	23.0 (7.0:65.0)
ApoB, mg/dL, mean (SD)	110.3 (42.0)	100.2 (41.4)	84.4 (37.7)	106.6 (40.3)	101.6 (41.5)

^a^ASCVD defined as CHD, ischemic stroke, or peripheral artery disease

^b^CHD defined according to the items pre-listed in the electronic case report form of the parent study, based on medical history and including adverse events observed during the parent study and during the pretreatment period of the OLE study

^c^Very high CV risk defined as patients with CHD or CHD risk equivalents according to 2016 European Society of Cardiology/European Atherosclerosis Society Guidelines [5]

^d^High CV risk defined as all other patients

*Apo*, apolipoprotein; *ASCVD*, atherosclerotic cardiovascular disease; *BMI*, body mass index; *CHD*, coronary heart disease; *CV*, cardiovascular, *HDL-C*, high-density lipoprotein cholesterol; *LDL-C*, low-density lipoprotein cholesterol; *Lp(a)*, lipoprotein(a); *ROW*, rest of world; *SD*, standard deviation

### Treatment Adherence

The median treatment duration during ODYSSEY OLE for this cohort was 131.7 weeks (range, 2.0–168.1 weeks). The mean treatment adherence to alirocumab was high across all regions, ranging from 97.7% (ROW) to 98.7% (Eastern Europe); overall, 99.2% of patients were ≥ 80% treatment adherent. Changes to background statin medication occurred in 21.9% of overall patients, including interruptions (8.0%), discontinuations (3.7%), change in statin type (4.4%), and dose increases (4.3%) or dose decreases (10.0%; Supplemental Table 1). Across the regions, there were fewer changes in background statin medication in Eastern Europe (13.3%) compared with other regions (Western Europe, 23.7%; North America, 21.0%; and ROW, 23.2%; Supplemental Table 1). Similarly, 20.7% of overall patients had a change in their background non-statin LLTs, including interruptions (5.2%), discontinuations (11.3%), change in LLT type (5.8%), dose increases (0.6%), and dose decreases (1.2%; Supplemental Table 2). Between the regions, there were fewer changes in non-statin LLTs in Eastern Europe and ROW (12.4% and 13.1%, respectively) compared with North America and Western Europe (22.4% and 23.9%, respectively; Supplemental Table 2).

### LDL-C Levels Used by Physicians for Alirocumab Dose Adjustment

A summary of patients with dose-adjustment decisions, and the LDL-C levels used to guide the physician dose-adjustment decisions, is provided in Table 2. Overall, irrespective of region, 72.9% of patients were maintained on alirocumab 75 mg Q2W; North America had the lowest proportion of patients with this dose decision compared with the other regions (59.2% versus 74.6–84.3%, respectively). The mean LDL-C at the time of decision to maintain the dose at 75 mg was approximately 1.8 mmol/L (70 mg/dL), with little variation across the regions (LDL-C at time of dose decision ranged from 1.7 to 2.0 mmol/L or 64.2 to 76.5 mg/dL; Table 2).

**Table 2:** Summary of patients with an alirocumab dose-adjustment decision during ODYSSEY OLE, and the LDL-C levels used to guide those shared physician–patient decisions, according to dose-adjustment decision and region (safety population)

Parameter	North America (*n =* 174)	Western Europe (*n =* 449)	Eastern Europe (*n =* 102)	ROW (*n =* 165)	All (*n* = 890)
Patients with any dose decision to maintain on 75 mg Q2W (%)	59.2	74.6	84.3	75.8	72.9
LDL-C at time of decision, mean (SD), mmol/L	1.9 (1.0)	1.7 (0.7)	2.0 (1.1)	1.9 (0.8)	1.8 (0.8)
Patients with any dose decision to increase to 150 mg Q2W (%)	56.3	41.2	28.4	44.8	43.4
LDL-C at time of decision, mean (SD), mmol/L	3.3 (1.2)	3.1 (1.3)	3.7 (1.3)	3.8 (1.5)	3.3 (1.3)
Patients with any dose decision to maintain on 150 mg Q2W (%)	98.9	97.8	100	100	98.6
LDL-C at time of decision, mean (SD), mmol/L	2.3 (1.3)	2.1 (1.3)	2.7 (1.5)	2.6 (1.3)	2.3 (1.3)

Patients who had alirocumab dose adjustment due to reasons other than LDL-C values were censored at the time before the corresponding dose adjustment. Dose adjustment from 150 to 75 mg Q2W was also possible during ODYSSEY OLE; however, due to the overall low number of these dose decisions (*n*/*N* [%] of 22/367 [6.0] for the overall population), these data are not presented here. Patients may be counted in more than one dose decision category

*LDL-C*, low-density lipoprotein cholesterol; *Q2W*, every 2 weeks; *ROW*, rest of world; *SD*, standard deviation

The proportion of patients with a dose decision to increase from alirocumab 75 to 150 mg Q2W ranged from 28.4% for Eastern Europe to 56.3% for North America and was 43.4% for the overall population (Table 2). Mean LDL-C values used by physicians for the decision to increase the dose from alirocumab 75 to 150 mg Q2W were higher in Eastern Europe and ROW (3.7 and 3.8 mmol/L; 144.0 and 145.2 mg/dL, respectively) compared with Western Europe and North America (3.1 and 3.3 mmol/L; 118.6 and 126.6 mg/dL, respectively; Table 2). Similarly, the mean LDL-C value at the time of dose decision to maintain on alirocumab 150 mg Q2W was higher in ROW and Eastern Europe (2.6 and 2.7 mmol/L; 100.3 and 102.6 mg/dL, respectively) compared with Western Europe and North America (2.1 and 2.3 mmol/L; 82.3 and 87.3 mg/dL, respectively; Table 2). Of the dose decisions to maintain at 150 mg Q2W, the proportion with LDL-C below 0.6 mmol/L (25 mg/dL) at the time of the decision was higher in North America, Western Europe, and Eastern Europe (ranging from 3.3 to 4.5%) compared with ROW (1.5%). Dose decrease from alirocumab 150 to 75 mg Q2W was also possible. However, as there were only a low number (22 decisions, with median [minimum:maximum] LDL-C at time of dose decision of 0.8 [0.2:2.4] mmol/L [30 (7.0:93.0) mg/dL] for the overall population), this was considered too few to analyze by region.

**Fig. 2:**
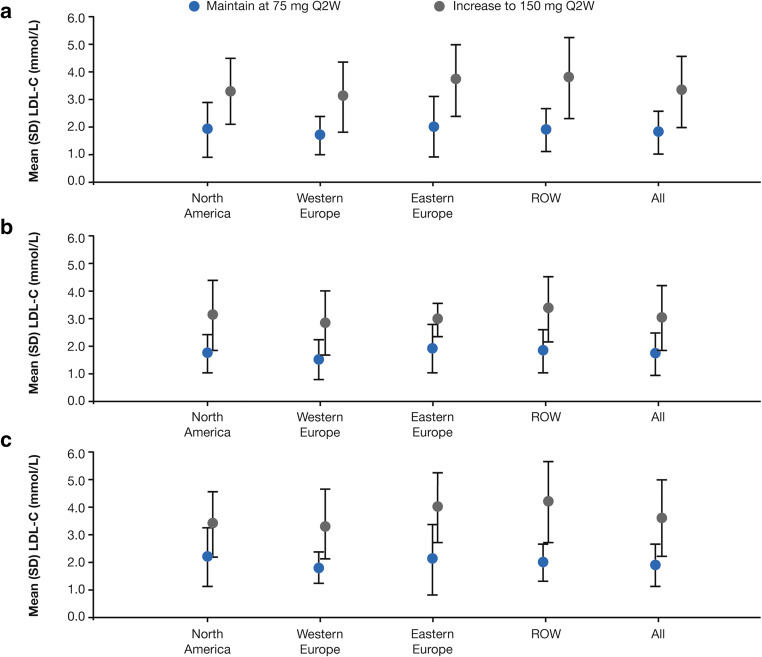
Summary of LDL-C levels used to guide dose decisions by region for **a** overall population, **b** patients with ASCVD at baseline, and **c** patients without ASCVD at baseline (safety population). ASCVD, atherosclerotic cardiovascular disease; LDL-C, low-density lipoprotein cholesterol; Q2W, every 2 weeks; ROW, rest of world; SD, standard deviation

When dose decisions were analyzed according to ASCVD status at baseline, the mean LDL-C value at the time of any dose decision was lower for patients with versus without ASCVD, both overall and across all regions (Fig. 2 and Supplemental Table 3). For patients without ASCVD, the mean LDL-C value at the time of decision to increase the alirocumab dose was lower in North America and Western Europe (both 3.4 mmol/L; 131 mg/dL) compared with Eastern Europe and ROW (4.0 and 4.2 mmol/L; 155.8 and 163.4 mg/dL, respectively). In addition, the proportion of patients without ASCVD with a dose decision to maintain on alirocumab 150 mg Q2W was higher in North America than in the other regions (50% versus 32.2–38.8%; Supplemental Table 3).

### Efficacy

At week 8, the mean ± standard deviation LDL-C was 2.2 ± 1.3 mmol/L (83.4 ± 50.3 mg/dL) for the overall population, a mean reduction of 1.7 mmol/L (64.1 mg/dL; 43.7%) from parent study baseline (Supplemental Fig. 1). Across the regions, mean LDL-C at week 8 ranged from 2.0 mmol/L (78.0 mg/dL) to 2.4 mmol/L (91.9 mg/dL; Supplemental Fig. 1B), or a mean percent reduction of 37.6 to 47.1% from parent study baseline (Supplemental Fig. 1A); the reductions in LDL-C were maintained through to week 96 of ODYSSEY OLE (Supplemental Fig. 1). For the overall population at week 96, the proportion of patients achieving LDL-C < 1.8 mmol/L (70 mg/dL) was 56.8% (Supplemental Fig. 2A), the proportion achieving LDL-C < 2.6 mmol/L (100 mg/dL) was 79.1% (Supplemental Fig. 2B), and the proportion achieving LDL-C < 1.8 mmol/L and/or ≥ 50% reduction from parent study baseline was 57.7% (Supplemental Fig. 2C). At week 96, the proportion of patients achieving LDL-C goals was lower in ROW compared with the other regions (46.7% versus 54.7–61.1% for LDL-C < 1.8 mmol/L, 71.1% versus 79.5–82.8% for LDL-C < 2.6 mmol/L, and 48.9% versus 54.7–61.9% for LDL-C < 1.8 mmol/L and/or ≥ 50% reduction from parent study baseline, respectively; Supplemental Fig. 2). The percentage change in LDL-C and other lipid parameters at week 96 of ODYSSEY OLE is presented both by region and overall in Supplemental Table 4.

### Safety

The safety of alirocumab during ODYSSEY OLE has been previously reported, both for the overall ODYSSEY OLE population [12] and for the current analysis population [13]. During ODYSSEY OLE, alirocumab was shown to have no unexpected long-term safety concerns.

## Discussion

Patients with HeFH are typically initiated on maximally tolerated statin therapy with or without ezetimibe, but in clinical practice, many patients do not reach their guideline-recommended LDL-C treatment goals without additional LLTs [4–6, 14–16]. The ODYSSEY OLE study was designed to evaluate the long-term efficacy and safety of alirocumab in patients with HeFH, as well as to investigate physician dosing decisions with the two alirocumab dosages available (75 and 150 mg Q2W), in a comparable setting to real-world clinical practice [12]. An analysis of ODYSSEY OLE showed that physicians considered the lower dose of alirocumab 75 mg Q2W to provide sufficient LDL-C lowering, without the need for dose increase, in more than half of patients who received starting dose alirocumab 75 mg Q2W [13]. This post hoc subanalysis has explored the regional variations in alirocumab dosing decisions made by physicians and their patients. A graphical overview of the study design and key findings is presented in Supplemental Fig. 3.

In the current analysis, regional differences in the pattern of physicians’ decisions to adjust the alirocumab dose were observed, with mean LDL-C values at the time of the decision to increase the dose from alirocumab 75 to 150 mg Q2W being higher in Eastern Europe and ROW (3.7 and 3.8 mmol/L; 144.0 and 145.2 mg/dL) than in Western Europe and North America (3.1 and 3.3 mmol/L; 118.6 and 126.6 mg/dL). Mean LDL-C values at the time of decision to maintain at alirocumab 150 mg Q2W were again higher in Eastern Europe and ROW (2.6 and 2.7 mmol/L; 102.6 and 100.3 mg/dL) compared with Western Europe and North America (2.1 and 2.3 mmol/L; 82.3 and 87.3 mg/dL). Although dose-adjustment decisions were primarily based on a patient’s LDL-C levels, differences in LDL-C-lowering approach could reflect variation in recognition or in levels of other atherogenic factors such as lipoprotein(a) [17]. Other factors could be regional differences in adherence to international guidelines and variations in national guidelines and local practices between countries.

The mean LDL-C at the time of decision to maintain the dose at alirocumab 75 mg Q2W was similar across the regions (ranging from 1.7 to 2.0 mmol/L; 64.2 to 76.5 mg/dL); however, North America had the lowest proportion of patients with this dose decision compared with the other regions (59.2% versus 74.6–84.3%, respectively). Conversely, North America had the highest proportion of patients with a dose decision to increase the alirocumab dose from 75 to 150 mg Q2W compared with the other regions (56.3% versus 28.4–44.8%, respectively).

These observed variations in clinical treatment decisions suggest that physicians may perceive the severity of HeFH and/or the treatment of HeFH differently depending on their region. Regional differences in treatment “traditions,” as well as the criteria utilized for the diagnosis of FH, may contribute to the differences in the perceived severity of FH. This, in turn, may be influenced by regional variations in clinical guidelines. For example, a recent comparison of six major dyslipidemia guidelines, covering North America [18, 19], Europe [5, 20, 21], and China [22], showed that, despite the overall similarities, differences did exist in terms of the classification of statin intensities, the use of risk estimators, the treatment of specific patient subgroups, and safety concerns [23]. Regional or country-specific clinical guidelines tend to align with the recommendations of international guidelines, for example, the South African dyslipidemia guideline consensus statement [24] was updated to reflect the ESC/EAS 2016 guideline [5] to ensure it was based on the most recent and best available evidence.

Although major dyslipidemia guidelines may be adopted on a regional basis, the implementation and achievement of LDL-C goals varies between regions. In the current analysis, LDL-C goal attainment was found to be lower in ROW compared with the other regions. The observational International ChoLesterol management Practice Study (ICLPS) investigated the achievement of LDL-C goals (according to the 2011 ESC/EAS guidelines) [25] in clinical practice in 18 countries outside Western Europe [26]. A subanalysis of ICLPS showed that LDL-C goal achievement rates were low in patients with familial hypercholesterolemia, even in patients receiving intensive LLTs [27]. In addition, a recent systematic review of patients with familial hypercholesterolemia in Latin America noted that patients in this region are undertreated and underdiagnosed according to major dyslipidemia guidelines, and recommended that establishing national guidelines for each population may improve diagnosis and management of patients [28].

A common feature of all current guidelines is the categorization of patients with HeFH and ASCVD as being at very high risk for future cardiovascular events; guidelines recommend a lower LDL-C goal for patients at very high cardiovascular risk compared with those at high cardiovascular risk. These recommendations are reflected in the current analysis, as the mean LDL-C at the time of any dose decision was lower for patients with versus without ASCVD, both overall and across all regions. As North America had a higher proportion of patients with ASCVD and also a higher proportion of those considered at very high cardiovascular risk, this may have contributed to the lower LDL-C values observed in North America at the time of the decision to increase the alirocumab dose compared with Eastern Europe and ROW (as noted above). It is interesting to note that, for patients without ASCVD, the mean LDL-C level at the time of decision to increase the dose of alirocumab was lower in North America and Western Europe (both 3.4 mmol/L) than in Eastern Europe and ROW (4.0 and 4.2 mmol/L). In addition, a higher proportion of patients without ASCVD were maintained on alirocumab 150 mg Q2W in North America than the other regions (50% versus 32.2–38.9%).

As well as differences in the interpretation and implementation of cholesterol guidelines in clinical practice, other factors should also be considered. For example, variations in the social determinants of health, access to resources required for the use of alirocumab, perception of very low LDL-C levels, and decisions by policy makers may all contribute to the regional differences in shared physician–patient decisions observed in the current analysis.

It is interesting to note that in the current analysis the achievement of LDL-C goals was lower in ROW than in the other regions. Possible reasons for this observation may include the underutilization of LLTs or higher LDL-C targets used in routine clinical practice in ROW countries compared with the other regions. Treatment underutilization has been previously observed with other LLTs in patients with HeFH. An earlier study conducted in The Netherlands reported that a common reason for not adopting maximum dose drug regimens was the acceptance by physicians of a higher than target LDL-C level. [29] A final note is that the ODYSSEY OLE trial was completed before the results from the large cardiovascular outcomes trials, FOURIER [30] and ODYSSEY OUTCOMES [31], were available, which have provided the best evaluation of the efficacy and safety of PCSK9 inhibitors. Therefore, although very low LDL-C levels are now known to present few additional safety issues, at the time of the current analysis, this was not well characterized. However, as both alirocumab dosages (alirocumab 75 mg and 150 mg Q2W) are approved for use [32, 33], and with alirocumab 75 mg Q2W as the recommended starting dose, the dose decisions observed in the current analysis are still clinically relevant.

Limitations of this analysis include the lack of a comparative control, the relatively small sample sizes when analyzed by region, and the possible introduction of bias due to the open-label study design. The small sample sizes also precluded the analysis of safety data by region. Furthermore, the interpretation of ROW data is limited as this category includes countries not related geographically (Fig. 1). Another consideration is that a single physician/study center may skew the observed regional variations; however, as there were multiple countries per region, and with most countries having more than one physician, only a minimal effect on results would be expected. Despite these limitations, the ODYSSEY OLE study assessed the efficacy and safety of alirocumab, as well as dose-adjustment decisions, over a median open-label treatment duration of 2.5 years in a relatively large population of patients with HeFH. [12]

In conclusion, for the management of hypercholesterolemia in patients with HeFH, regional differences in physician treatment decisions were observed. These regional variations may reflect differences in how physicians perceive the severity of HeFH, influenced by both regional and international clinical guidelines, as well as differences in patient characteristics particular to their clinical practice. A greater understanding of these differences is important to enable a consistent approach to the management of hypercholesterolemia in patients with HeFH.

## Electronic Supplementary Material

ESM 1 (DOCX 1222 kb)

## Data Availability

Qualified researchers may request access to patient-level data and related study documents, including the clinical study report, study protocol with any amendments, blank case report form, statistical analysis plan, and data set specifications. Patient-level data will be anonymized, and study documents will be redacted to protect the privacy of trial participants. Further details on Sanofi’s data sharing criteria, eligible studies, and process for requesting access can be found at https://www.clinicalstudydatarequest.com.
