# Supplementary material for: Regional Variations in Alirocumab Dosing Patterns in Patients with Heterozygous Familial Hypercholesterolemia During an Open-Label Extension Study
Source: Cardiovasc Drugs Ther. 2020 May 4;34(4):515–23. doi: 10.1007/s10557-020-06984-0 (PMC7334259; doi:10.1007/s10557-020-06984-0)
Supplement: Supplementary file 1 — (DOCX 1222 kb) [file 10557_2020_6984_MOESM1_ESM.docx]

Supplemental Table 1. Summary of adjustments to background statin therapy, and the reasons for those adjustments, made during ODYSSEY OLE by region (safety population)

| ***n* (%)** | **North America** **(*n =* 176)** | **Western Europe** **(*n =* 460)** | **Eastern Europe** **(*n =* 105)** | **ROW** **(*n =* 168)** | **All** **(*n* = 909)** |
| --- | --- | --- | --- | --- | --- |
| Any change in statin**^a^** | 37 (21.0) | 109 (23.7) | 14 (13.3) | 39 (23.2) | 199 (21.9) |
| Statin interruption | 22 (12.5) | 33 (7.2) | 3 (2.9) | 15 (8.9) | 73 (8.0) |
| Reason for statin interruption |  |  |  |  |  |
| Adverse event | 11 (6.3) | 14 (3.0) | 1 (1.0) | 4 (2.4) | 30 (3.3) |
| LDL-C too low^b^ | 0 (0.0) | 3 (0.7) | 1 (1.0) | 0 (0.0) | 4 (0.4) |
| Poor adherence | 1 (0.6) | 10 (2.2) | 1 (1.0) | 3 (1.8) | 15 (1.7) |
| Statin discontinuation | 12 (6.8) | 11 (2.4) | 3 (2.9) | 8 (4.8) | 34 (3.7) |
| Reason for statin discontinuation |  |  |  |  |  |
| Adverse event | 6 (3.4) | 7 (1.5) | 1 (1.0) | 4 (2.4) | 18 (2.0) |
| LDL-C too low^b^ | 0 (0.0) | 2 (0.4) | 1 (1.0) | 0 (0.0) | 3 (0.3) |
| Poor adherence | 0 (0.0) | 1 (0.2) | 1 (1.0) | 0 (0.0) | 2 (0.2) |
| Change in statin type | 7 (4.0) | 17 (3.7) | 2 (1.9) | 14 (8.3) | 40 (4.4) |
| Reason for change in statin type |  |  |  |  |  |
| Adverse event | 4 (2.3) | 10 (2.2) | 2 (1.9) | 3 (1.8) | 19 (2.1) |
| Statin dose increase | 5 (2.8) | 21 (4.6) | 6 (5.7) | 7 (4.2) | 39 (4.3) |
| Reason for statin dose increase |  |  |  |  |  |
| LDL-C too high^b^ | 2 (1.1) | 7 (1.5) | 4 (3.8) | 1 (0.6) | 14 (1.5) |
| Other | 3 (1.7) | 10 (2.2) | 2 (1.9) | 4 (2.4) | 19 (2.1) |
| Statin dose decrease | 11 (6.3) | 59 (12.8) | 10 (9.5) | 11 (6.5) | 91 (10.0) |
| Reason for statin dose decrease |  |  |  |  |  |
| Adverse event | 5 (2.8) | 13 (2.8) | 2 (1.9) | 3 (1.8) | 23 (2.5) |
| LDL-C too low^b^ | 3 (1.7) | 36 (7.8) | 7 (6.7) | 1 (0.6) | 47 (5.2) |
| Poor adherence | 0 (0.0) | 0 (0.0) | 1 (1.0) | 0 (0.0) | 1 (0.1) |
| Other | 2 (1.1) | 8 (1.7) | 1 (1.0) | 2 (1.2) | 13 (1.4) |

^a^Data include any changes in dose, type or discontinuation at any visit after OLE entry, compared with previous recording.

^b^As per investigator judgment.

LDL-C, low-density lipoprotein cholesterol; ROW, rest of world.

Supplemental Table 2. Summary of adjustments to background non-statin LLTs, and the reasons for those adjustments, made during ODYSSEY OLE by region (safety population)

| ***n* (%)** | **North America** **(*n =* 176)** | **Western Europe** **(*n =* 460)** | **Eastern Europe** **(*n =* 105)** | **ROW** **(*n =* 168)** | **All** **(*n =* 909)** |
| --- | --- | --- | --- | --- | --- |
| Any change in non-statin LLT**^a^** | 43 (24.4) | 110 (23.9) | 13 (12.4) | 22 (13.1) | 188 (20.7) |
| Non-statin LLT interruption | 9 (5.1) | 31 (6.7) | 4 (3.8) | 3 (1.8) | 47 (5.2) |
| Reason for non-statin LLT interruption |  |  |  |  |  |
| LDL-C too low^b^ | 0 (0.0) | 14 (3.0) | 2 (1.9) | 1 (0.6) | 17 (1.9) |
| Supply issue | 3 (1.7) | 4 (0.9) | 1 (1.0) | 1 (0.6) | 9 (1.0) |
| Poor adherence | 2 (1.1) | 3 (0.7) | 1 (1.0) | 1 (0.6) | 7 (0.8) |
| Non-statin LLT discontinuation | 37 (21.0) | 45 (9.8) | 6 (5.7) | 15 (8.9) | 103 (11.3) |
| Reason for non-statin LLT discontinuation |  |  |  |  |  |
| Adverse event | 4 (2.3) | 7 (1.5) | 1 (1.0) | 2 (1.2) | 14 (1.5) |
| LDL-C too low^b^ | 9 (5.1) | 30 (6.5) | 5 (4.8) | 5 (3.0) | 49 (5.4) |
| Change in non-statin LLT type | 4 (2.3) | 41 (8.9) | 2 (1.9) | 6 (3.6) | 53 (5.8) |
| Reason for change in non-statin LLT type |  |  |  |  |  |
| LDL-C too high^b^ | 3 (1.7) | 3 (0.7) | 1 (1.0) | 2 (1.2) | 9 (1.0) |
| Other | 0 (0.0) | 37 (8.0) | 1 (1.0) | 4 (2.4) | 42 (4.6) |
| Non-statin LLT dose increase | 1 (0.6) | 3 (0.7) | 1 (1.0) | 0 (0.0) | 5 (0.6) |
| Reason for non-statin LLT dose increase |  |  |  |  |  |
| LDL-C too high^b^ | 0 (0.0) | 3 (0.7) | 1 (1.0) | 0 (0.0) | 4 (0.4) |
| Non-statin LLT dose decrease | 3 (1.7) | 7 (1.5) | 1 (1.0) | 0 (0.0) | 11 (1.2) |
| Reason for non-statin dose decrease |  |  |  |  |  |
| Poor adherence | 0 (0.0) | 0 (0.0) | 1 (1.0) | 0 (0.0) | 1 (0.1) |

^a^Data include any changes in dose, type or discontinuation at any visit after OLE entry, compared with previous recording.

^b^As per investigator judgment.

LDL-C, low-density lipoprotein cholesterol; LLT, lipid-lowering therapy; ROW, rest of world.

Supplemental Table 3. Summary of patients with an alirocumab dose adjustment decision during ODYSSEY OLE, and the LDL-C levels used to guide those shared physician–patient decisions, by dose adjustment decision and region according to ASCVD status at OLE baseline (safety population)

| **Patients with ASCVD** | **North America** | | **Western Europe** | | **Eastern Europe** | | **ROW** | | **All** | |
| --- | --- | --- | --- | --- | --- | --- | --- | --- | --- | --- |
|  | **Yes** **(*n =* 96)** | **No** **(*n =* 80)** | **Yes** **(*n =* 224)** | **No** **(*n =* 236)** | **Yes** **(*n =* 51)** | **No** **(*n =* 54)** | **Yes** **(*n =* 78)** | **No** **(*n =* 90)** | **Yes** **(*n =* 449)** | **No** **(*n =* 460)** |
| Patients with any dose decision to maintain on 75 mg Q2W, n (%) | 55  (57.3) | 48 (60.0) | 151 (67.4) | 174 (73.7) | 41 (80.4) | 43 (79.6) | 60 (76.9) | 63 (70.0) | 307 (68.4) | 328 (71.3) |
| LDL-C at time of decision, mean (SD), mmol/L | 1.7 (0.7) | 2.2 (1.1) | 1.5 (0.7) | 1.8 (0.6) | 1.9 (0.9) | 2.1 (1.3) | 1.8 (0.8) | 2.0 (0.7) | 1.7 (0.8) | 1.9 (0.8) |
| Patients with any dose decision to increase to 150 mg Q2W, n (%) | 54 (56.3) | 43 (53.8) | 101 (45.1) | 83 (35.2) | 8 (15.7) | 21 (38.9) | 36 (46.2) | 38 (42.2) | 199 (44.3) | 185 (40.2) |
| LDL-C at time of decision, mean (SD), mmol/L | 3.1 (1.3) | 3.4 (1.2) | 2.8 (1.2) | 3.4 (1.3) | 2.9 (0.6) | 4.0 (1.3) | 3.3 (1.2) | 4.2 (1.5) | 3.0 (1.2) | 3.6 (1.4) |
| Patients with any dose decision to maintain on 150 mg Q2W, n (%) | 50 (52.1) | 40 (50.0) | 101 (45.1) | 76 (32.2) | 8 (15.7) | 19 (35.2) | 33 (42.3) | 35 (38.9) | 192 (42.8) | 170 (37.0) |
| LDL-C at time of decision, mean (SD), mmol/L | 2.1 (1.4) | 2.4 (1.2) | 1.9 (1.0) | 2.5 (1.5) | 2.2 (0.7) | 2.9 (1.6) | 2.1 (1.1) | 3.0 (1.4) | 2.0 (1.1) | 2.6 (1.4) |

ASCVD, atherosclerotic cardiovascular disease; LDL-C, low-density lipoprotein cholesterol; Q2W, every 2 weeks; ROW, rest of world; SD, standard deviation.

Supplemental Table 4. Percent changes in lipid parameters from parent study baseline to Week 96 of ODYSSEY OLE (modified intention-to-treat analysis)

| **Change from parent study baseline, %** | **North America** | **Western Europe** | **Eastern Europe** | **ROW** | **All** |
| --- | --- | --- | --- | --- | --- |
| LDL-C, mean (SD) [*n*] | −46.8 (24.7) [64] | −49.9 (26.0) [378] | −44.4 (27.9) [83] | −44.0 (29.9) [135] | −47.7 (27.0) [660] |
| Non-HDL-C, mean (SD) [*n*] | −40.4 (26.5) [64] | −42.4 (23.9) [381] | −34.8 (29.0) [87] | −36.1 (28.3) [137] | −39.9 (25.9) [669] |
| ApoB, mean (SD) [*n*] | −38.7 (22.0) [63] | −39.4 (20.8) [376] | −34.1 (25.0) [87] | −33.4 (24.9) [135] | −37.4 (22.5) [661] |
| Lp(a), median (Q1:Q3) [*n*] | −30.2 (−45.5:−16.7) [63] | −25.0 (−42.8:−6.5) [377] | −28.6 (−50.0:−2.9) [87] | −32.7 (−45.5:−12.5) [135] | −26.7 (−45.0:−9.1) [662] |
| Fasting TGs, median (Q1:Q3) [*n*] | −5.7 (−29.4:21.8) [64] | −1.7 (−23.1:27.1) [379] | 1.6 (−18.7:33.3)  [86] | 1.9 (−19.3:40.5) [137] | −1.2 (−21.4:29.8) [666] |
| HDL-C, mean (SD) [*n*] | 6.5 (20.2) [64] | 7.7 (18.7) [381] | 5.1 (15.4) [87] | 6.3 (19.6) [137] | 7.0 (18.6) [669] |
| Total cholesterol, mean (SD) [*n*] | −30.0 (21.7) [64] | −30.8 (18.8) [381] | −24.7 (21.2) [87] | −26.8 (22.7) [137] | −29.1 (20.3) [669] |

Apo, apolipoprotein; HDL-C, high-density lipoprotein cholesterol; LDL-C, low-density lipoprotein cholesterol; Lp(a), lipoprotein (a); ROW, rest of world; SD, standard deviation; TGs, triglycerides.

Supplemental Fig. 1. (A) Percent change in calculated LDL-C from baseline of parent studies to Week 8 and Week 96 of ODYSSEY OLE, and (B) mean (SE) LDL-C levels over time at Week 96, according to region and overall (modified intention-to-treat analysis)

(A)


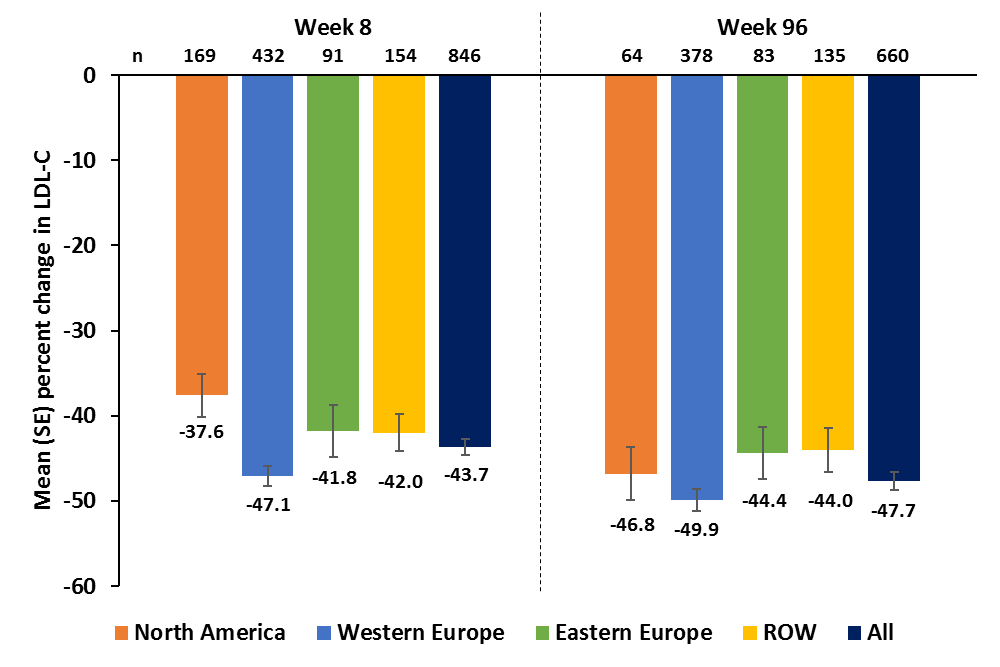


(B)


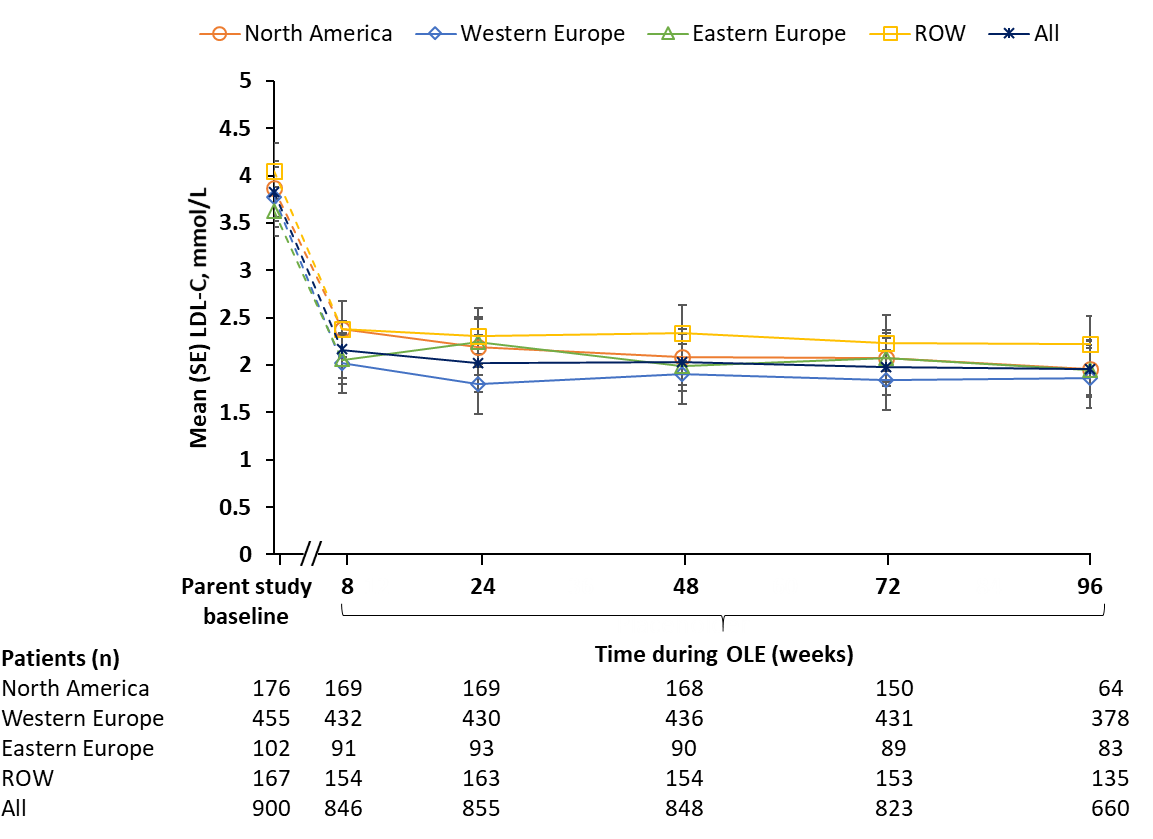


LDL-C, low-density lipoprotein cholesterol; ROW, rest of world; SE, standard error.

Supplemental Fig. 2. Proportion of patients achieving LDL-C goals by region during ODYSSEY OLE. (A) LDL-C <1.8 mmol/L (70 mg/dL), (B) LDL-C <2.6 mmol/L (100 mg/dL), and (C) LDL-C <1.8 mmol/L (70 mg/dL) and/or ≥50% reduction in LDL‑C from parent study baseline (modified intention-to-treat analysis)^a^

(A)


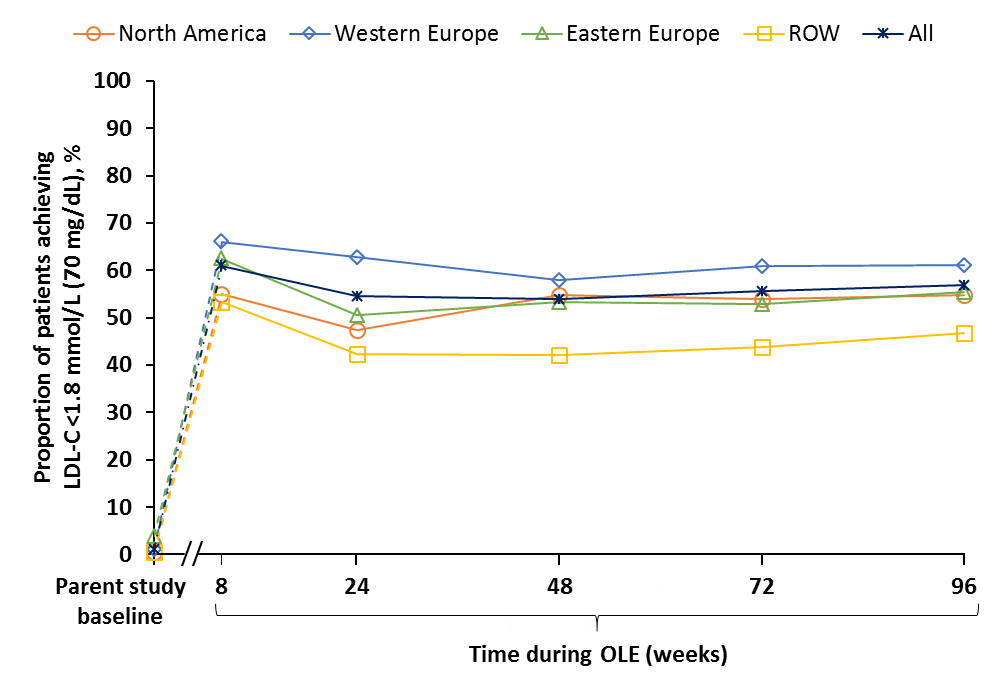


(B)


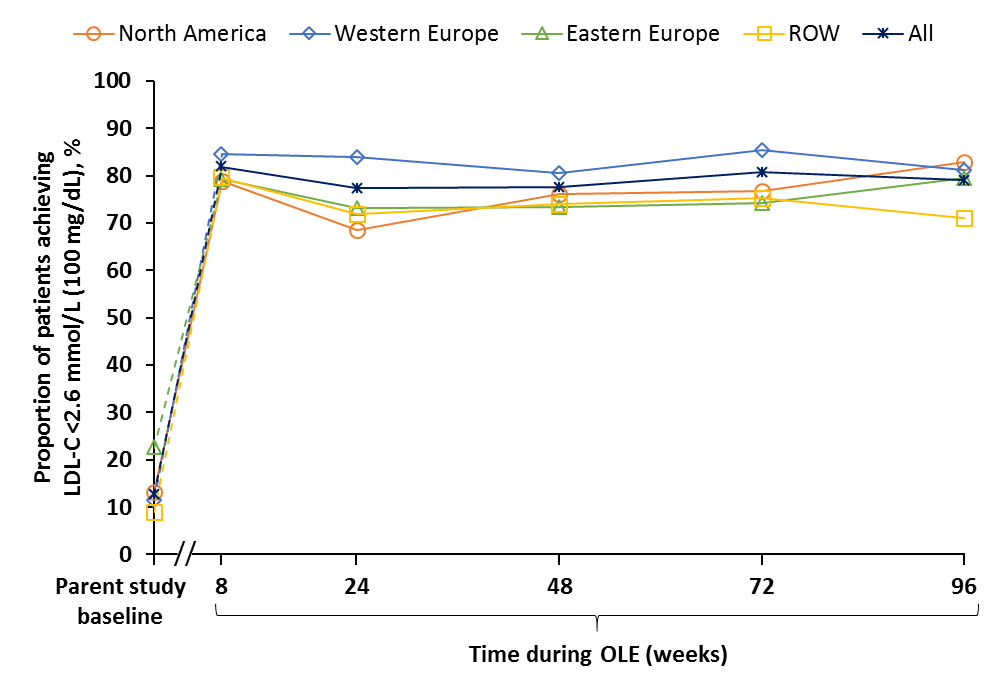


(C)


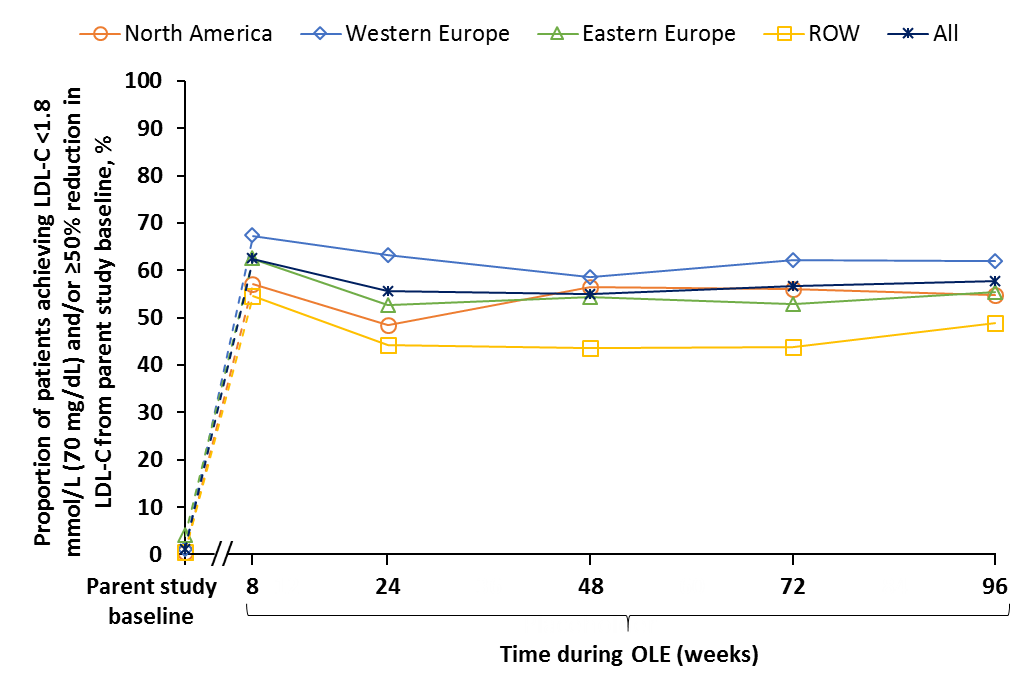


^a^Results are reported up to Week 96 due to a decrease in patient numbers after this time point.

LDL-C, low-density lipoprotein cholesterol; ROW, rest of world.

Supplemental Fig 3. Graphical summary of the ODYSSEY OLE study design and key results


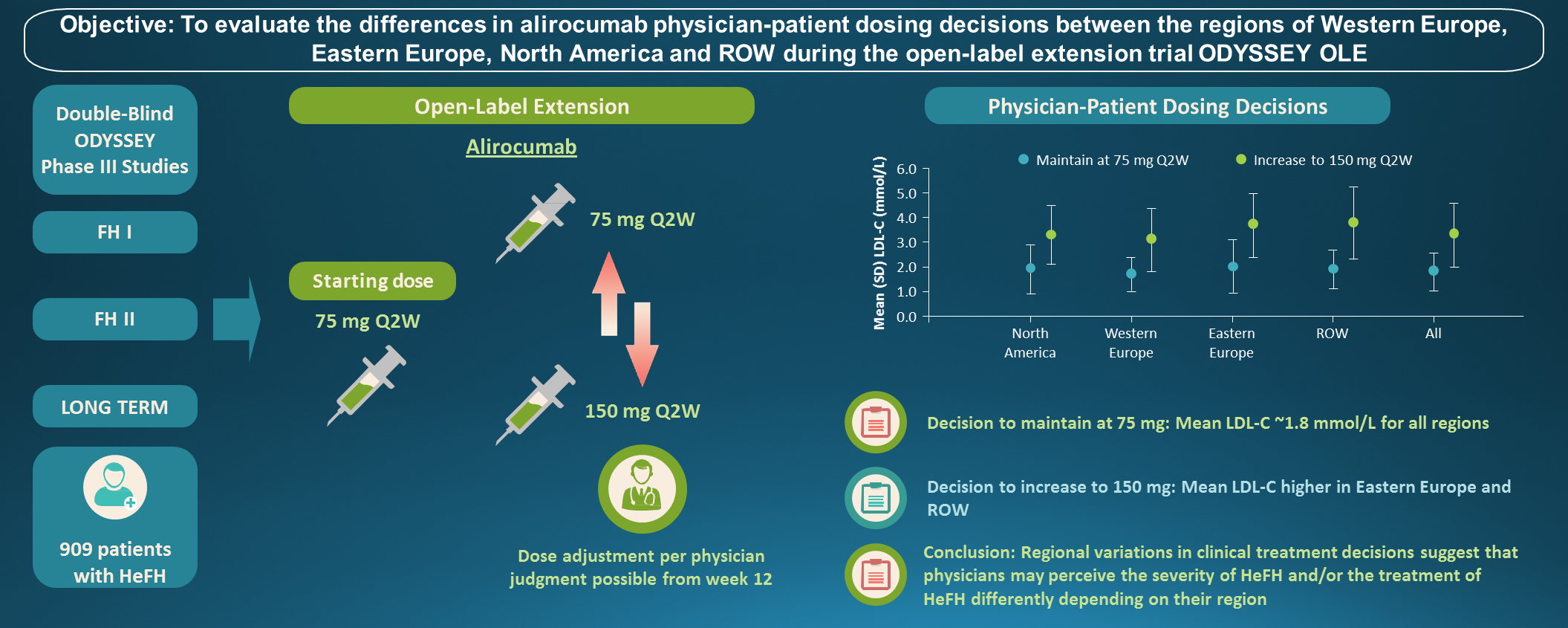


HeFH, heterozygous familial hypercholesterolemia; LDL-C, low-density lipoprotein cholesterol; Q2W, every 2 weeks; ROW, rest of world; SD, standard deviation.
